# The promising potential of polynucleotide injections for knee osteoarthritis: A systematic review of literature and meta‐analysis of randomized control trials

**DOI:** 10.1002/jeo2.70428

**Published:** 2025-09-15

**Authors:** Joseph Elphingstone, Michael Bowler, Pietro Conte, Giuseppe Anzillotti, Elizaveta Kon

**Affiliations:** ^1^ Phoebe Putney Memorial Hospital Albany Georgia USA; ^2^ Department of Sports and Exercise Medicine University of Alabama at Birmingham Birmingham Alabama USA; ^3^ Medical College of Georgia Augusta Georgia USA; ^4^ IRCCS Humanitas Research Hospital Rozzano Milan Italy; ^5^ Department of Biomedical Sciences Humanitas University Pieve Emanuele Milan Italy

**Keywords:** hyaluronic acid, knee, osteoarthritis, polynucleotides, viscosupplement

## Abstract

**Purpose:**

The present study aims to synthesize and evaluate the evidence on the effectiveness of intra‐articular polynucleotides (PNs) for knee osteoarthritis (OA).

**Methods:**

In March 2024, we searched MEDLINE, Emcare, Web of Science and Cochrane Library for studies involving PNs for knee OA in human subjects. Inclusion criteria consisted of human subjects with knee OA and the use of intra‐articular PNs. Studies were excluded if they were basic science, included non‐knee OA pathology, did not use PNs, were case reports, or if there was no available full‐text article. The primary outcome was pain, and secondary measures were functional outcome scores and adverse events. Risk of bias was analyzed using Cochrane's Risk of Bias Tool for randomized controlled trials and ROBINS‐I for non‐randomized studies.

**Results:**

Twelve studies were included for systematic review and five for meta‐analysis. Two studies evaluated a combined treatment of PN and HA (PNHA) to HA but were included only for qualitative analysis. Meta‐analysis demonstrated significantly better pain improvement in PN than HA at 2 months (mean difference [MD] = −1.04 [−1.63 to −0.45], *p* = 0.0006) and four months (MD = −0.84 [−1.45 to −0.24], *p* = 0.006). Functional outcomes also favoured PNs at 2 months (standardized mean difference [SMD] = 0.46 [0.17–0.74], *p* = 0.002) and 4 months (SMD = 0.25 [0.00–0.50], *p* = 0.05) over HA. Data from PNHA studies suggested better pain and functional outcomes than those from HA. Adverse events with PNs were not significantly different from those receiving HA (RR = 1.97 [0.72–5.37], *p* = 0.187).

**Conclusion:**

PN injections are a safe and more effective option for treating knee OA than conventional HA. Treatments combining PN and HA also appear to be a promising therapeutic option. However, limited statistical power and potential publication bias warrant further research to enhance confidence in these findings.

**Level of Evidence:**

Level IV, systematic review of Levels II–IV studies. Level I, meta‐analysis of Level I studies.

**Abbreviations**
BMI: body mass index
HA: hyaluronic acid
HMWHA: high molecular weight hyaluronic acid
KL: Kellgren–Lawrence
KOOS: Knee injury and Osteoarthritis Outcome Score
KSS: Knee Society Score
LMWHA: low molecular weight hyaluronic acid
MCII: minimally clinically important improvement
MD: mean difference
OA: osteoarthritis
PN: polynucleotide
PNHA: polynucleotide plus hyaluronic acid
PROM: patient‐reported outcome measure
RCT: randomized controlled trial
RR: relative risk
SMD: standardized mean difference
VAS: visual analogue scale
WOMAC: Western Ontario and McMaster University Osteoarthritis Index

## INTRODUCTION

Knee osteoarthritis (OA) is one of the most prevalent and debilitating conditions worldwide, affecting more than 650 million patients and incurring more than 27 billion USD in medical expenditures annually [2, 5]. OA management is primarily symptomatic and includes various treatments, such as nonsteroidal anti‐inflammatory drugs (NSAIDs), physical therapy and injectable therapies to temporarily alleviate symptoms and delay definitive management with arthroplasty. One of the mainstay injectables for OA is hyaluronic acid (HA). HA is a naturally occurring component of synovial fluid that adds to its rheologic properties and provides anti‐inflammatory effects [1, 8]. Viscosupplementation with HA is cost‐effective, reducing overall OA medical expenses and delaying the need for arthroplasty by an average of 15 months [21]. Despite the widespread use of HA, its efficacy remains modest and inconsistent, especially in patients with moderate to severe OA.

In recent years, polynucleotides (PNs) have emerged as a promising alternative to HA for viscosupplementation. Initially developed for aesthetic medicine, PNs are a mixture of naturally occurring DNA‐derived macromolecules of different lengths. In the orthopaedic setting, most evidence relies on the PNs obtained from trout gonads through High Purification Technology (HPT) developed by Mastelli srl, but PNs can originate from a variety of sources using different procedures [3]. Made from long polymers of nucleic acids, they exhibit non‐Newtonian properties, giving them viscosity and elasticity similar to those of HA [23]. Hence, PNs are able to restore the viscoelasticity of synovial fluid by providing a scaffold and are also able to promote the restoration of a physiological microenvironment and the development of healthy cartilage. They favour the recovery of chondrocyte vitality with the physiologic deposition of cartilage matrix [9, 15, 20, 29]. Given the limitations of current non‐cellular injectables, there is a clinical need to evaluate alternative therapies with potential biological benefits.

With the promising basic science evidence for PNs over the past decade, a growing body of clinical literature has emerged evaluating the efficacy of intra‐articular PNs injections in treating knee OA. Recent clinical trials have produced mixed conclusions, with several studies showing significant improvements in pain relief and functional outcomes with PN injections compared to traditional HA treatments. With this accumulating evidence, the aim of this systematic review and meta‐analysis of randomized controlled trials (RCTs) is to gather and critically assess all available evidence on the use of PNs for the symptomatic treatment of knee OA.

## METHODS

This study followed the guidelines of the Preferred Reporting Items for Systematic Reviews and Meta‐Analysis 2020 statement.

### Information sources

This study utilized multiple databases, including MEDLINE, Embase, Web of Science and Cochrane Library. The Database search was performed in March 2024. There were no restrictions on the publication date. The terms (MeSH ‘Osteoarthritis’ OR title/abstract keywords ‘arthritis’, ‘osteoarthritis’, ‘osteoarthrosis’, ‘gonarthrosis’, ‘gonoarthritis’) AND (MESH ‘Polynucleotides’ OR title/abstract keywords ‘polynucleotide’,* ‘PN’) were queried for MEDLINE, Emcare, and Cochrane Library. MeSH search was replaced with Topic Search for Web of Science. Additional articles were manually incorporated after reviewing in‐article citations for included studies. This study protocol was registered on PROSPERO (registration number CRD42025633341).

### Eligibility criteria

Two authors independently reviewed the initial study pool generated from the search query. The initial review screened article titles and abstracts that included the terms ‘polynucleotides’ and ‘osteoarthritis’. Full‐text reviews assessed whether studies evaluated human subjects treated with polynucleotides approved for intra‐articular use. Studies needed to obtain at least one patient‐reported outcome measure (PROM), such as the visual analogue scale (VAS), Knee Society Score (KSS), Knee injury and Osteoarthritis Outcome Score (KOOS) or Western Ontario and McMaster University Osteoarthritis Index (WOMAC). We also evaluated adverse outcomes from injections. We excluded basic science studies that assessed non‐human subjects or human studies with non‐knee OA pathology, did not use PNs as a treatment, case reports, and studies without an available full‐text article. We also excluded studies that did not use PN injections not approved for intra‐articular application. If there was uncertainty regarding whether a study should be included, full‐length articles were assessed for eligibility. Any discrepancies in the final article selection were discussed among authors (J.E. and M.B.).

### Risk of bias assessment

Two reviewers (J.E. and M.B.) independently assessed the quality of the RCT study using Cochrane's Risk of Bias Tool [13]. Each study was appraised based on the randomization process, deviation from intended intervention, missing outcome data, outcome measurement and reported result selection. The domains were scored as having either low, some concern or a high risk of bias. Discrepancies in bias analyses were resolved with discussion until a consensus was reached. Similarly, bias was evaluated in non‐RCT studies with ROBINS‐I tool [32].

### Statistical analysis

The primary outcome of this study was to investigate changes in reported pain, demonstrated by VAS scores, among RCTs. Secondary outcomes were related to changes in function, which were assessed by a variety of PROMs, including KSS, KOOS and WOMAC. Additionally, patient demographics (age, sex and OA severity), injectate characteristics, adverse events, and serum and synovial fluid biomarker data were tabulated and included within the study's descriptive statistics. If the means and standard deviations (SDs) were not explicitly listed in text or on tables, the corresponding authors were contacted for this data. If authors could not be contacted, WebPlotDigitizer v4.7 was used to quantify line and bar graphs with error bars. SDs for box and whisker plots were estimated based on the Wan et al. formula ([Q3–Q1]/1.35), assuming normally distributed data [34]. Meta‐Mar v4.0.2 software was used for meta‐analysis and forest plot generation. *I* ^2^ statistic was used to assess heterogeneity and guide the use of common effects (low heterogeneity, *I* ^2^ < 0.5) versus random effects models for (high heterogeneity, *I* ^2^ ≥ 0.5). We used a common effects model using restricted maximum‐likelihood (REML) to estimate study variance. This method is useful when dealing with models that have both fixed and random effects. We utilized a mean difference and a 95% confidence interval (CI) for our primary pain outcome. Standard mean difference was calculated with a random‐effects model for functional outcome measure analysis, as papers utilized different PROMs. Relative risk (RR) ratios were calculated based on reported adverse events from all studies with a control group, utilizing inverse variance pooling methods and REML for heterogeneity estimation. A Funnel plot was also generated using this software to assess publication bias using the pain outcome data reported by the RCTs at 1, 2 and 4 months. The inverse variance method was used to create the funnel plot, as it reduces variability in the pooled effect estimates.

## RESULTS

**Figure 1:**
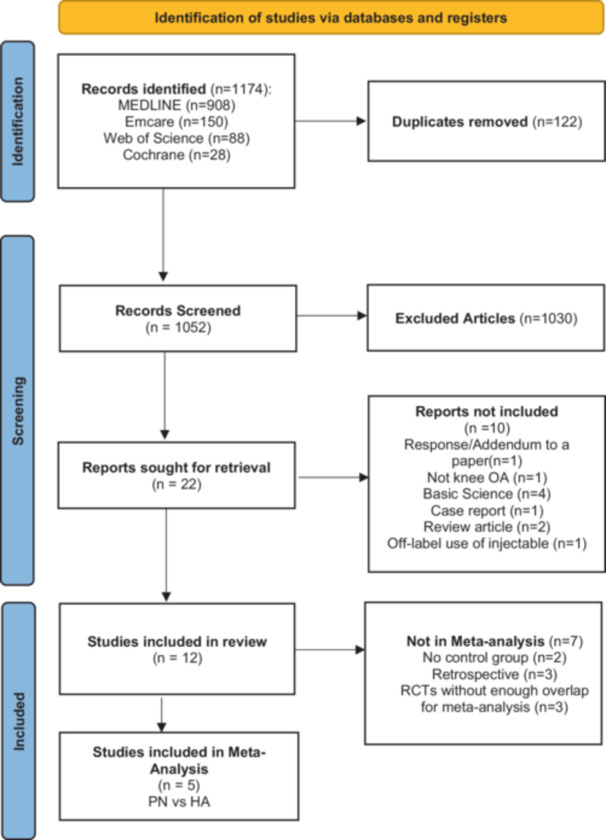
PRISMA flowchart of the literature search. HA, hyaluronic acid; PN, polynucleotide; PRISMA, Preferred Reporting Items for Systematic Reviews and Meta‐Analysis; RCT, randomized controlled trial.

The initial search yielded 1174 articles. After duplicate removal, 1052 underwent title and abstract review and 22 for subsequent full‐length review. After the full‐length review, 12 were included in the systematic review and five in the meta‐analysis (Figure 1). For a summary of study qualities, refer to Table 1. The articles included seven RCTs, three retrospective cohorts, one prospective case series, and one observational study. Two different study treatment strategies were assessed: PNs alone compared to HA, or a combination of PN and HA (PNHA) compared to HA. The systematic review includes a population of 1068 study patients (781 PN, 50 PNHA and 237 HA), with an average age of 65 years (range: 18–82), body mass index (BMI) of 27.1 (17.1–36.1), and 67.8% were female. The study by Stagni et al. [31] is a 2‐year follow‐up analysis from an earlier paper published by Dallari et al. [6]; thus, the redundant demographic data were not included. The meta‐analysis cohort consists of 281 patients (140 PN and 141 HA) with an average age of 64 years (31‐82), BMI of 28.1 (23.6‐36.1), and 64.8% were female. Nine studies [6, 11, 14, 16, 18, 19, 22, 25, 31] described OA classification (Kellgren–Lawrence [KL] Grade I–IV), with only four [14, 18, 19, 25] reporting the frequency of each OA grade. Most patients had mild (Grade II, 46.6%) or moderate (Grade III, 33.6%) OA. Of the 12 included studies, only 1 [24] incorporated physical therapy as part of post‐injection management. However, the group did not describe a specific protocol.

**Table 1:** Characteristics of the studies included in the systematic review.

Study	Level of evidence	Number of patients	Number of injections	Product description (company)	Gender (M/F)	Age, SD (range)	BMI, SD (range)	OA severity (%)	PROMs assessed	Time points	Adverse outcomes	Main findings
Vanelli 2010 [33]	Randomized, double‐blind clinical trial	PN 30	5	Condrotide (Mastelli srl, Sanremo, Italy)	10/19	60 (37–79)	26.7 (23.9–36.1)	NA	VAS, KOOS	1, 2, 4 months	PN: Knee pain (*n* = 1) HA: None	No significant difference in pain or function at any time point
		HA 30	5	Sinovial (HMWHA + LMWHA) (Laboratoires Genevrier, Antibes, France)	10/20	67 (46–82)	28.8 (20.9–35.7)	NA				
Meccariello 2013 [22]	Retrospective cohort	PN 30	3	NA	15/15	70.6 (55–78)	25.7 (22.4–29.2)	II: 30	VAS, KOOS	1, 3, 4 months	PN: None HA: None	No difference in pain until six months, which was significantly lower with PN No functional difference at any time point
		HA 30	3	NA	15/15	70.4 (55–75)	24.6 (21.2–27.3)	II: 30				
Zazgyva 2013 [36]	Randomized, double‐blind clinical trial	PN 15	3	Condrotide (Mastelli srl, Sanremo, Italy)	7/8	62 (31–71)	27.7 (23.6–33.2)	NA	VAS, KOOS, KSS	1, 2, 4 months	PN: Knee pain (*n* = 2) HA: Knee pain (*n* = 1)	Significantly better pain and function (KSS) than HA
		HA 15	3	Synocrom (HMWHA) (Croma Pharma, Leobendorf, Austria)	6/9	60 (18–68)	29.1 (24.2–36.1)	NA				
Giarratana 2014 [10]	Randomized, double‐blind, parallel‐group clinical trial	PN 36	3	Condrotide (Mastelli srl, Sanremo, Italy)	16/20	64.9 (31–80)	NA	NA	VAS, KOOS	1, 2, 4, 6 months	PN: None HA: None	No difference in pain or function between groups Earlier improvement in KOOS symptoms in PN than HA
		HA 36	3	Hyalubrix (HMWHA) (Fidia Farmaceutici, Abano Terme, Italy)	15/21	64.1 (43–76)	NA	NA				
Guelfi 2020 [11]	Retrospective historical case control	PN 11	3	Condrotide (Mastelli srl, Sanremo, Italy)	NA	NA	NA	I–IV	WOMAC	1, 3, 6 months	PN: Knee pain and swelling (*n* = 4) HA: Not assessed	Similar reduction in WOMAC scores between PN and HA
		HA 11	3	‘MMWHA’	NA	NA	NA	I–IV				
Kim 2021 [16]	Retrospective case control	PN 5	3	Conjuran (PharmaResearch Co. Ltd, Gangneung, South Korea)	1/4	68.0 ± 6.4	24.3 (NA)	I–III	VAS, WOMAC, EQ‐5D, K‐PDQ	1 month	PN: None HA: None	Significantly better pain relief with PN than HA at one month No difference for functional outcome measures
		HA 5	3	Hyruan Plus (HMWHA) (LG Life Sciences, Boston, USA)	0/5	68.4 ± 8.2	26.8 (NA)	I–III				
Jang 2022 [14]	Prospective case series	PN 51	5	HP cell Vitaran J (BRPHARM Co., Wonju, South Korea)	14/37	62.7 ± 8.9 (44–77)	25.6 ± 3.2 (17.1–27.7)	I: 28 (54.9) II:19 (37.3) III: 4 (7.8)	VAS, K‐WOMAC, BSV	3 Months	Not assessed	Significantly improved VAS and WOMAC at one and four months Possible increase in Subchondral bone
		No Control	–	–	–	–	–	–				
Moon 2023 [25]	Randomized, double‐blind, parallel‐group clinical trial	PN 30	3	Conjuran (PharmaResearch Co. Ltd, Gangneung, South Korea)	11/19	67.5 ± 7.0	NA	I: 6 (24) II: 11 (44) III: 8 (32)	VAS, WOMAC	2, 4 months	PN: Knee pain (*n* = 2), knee swelling (*n* = 1) HA: Knee pain (*n* = 2)	PN provided better pain reduction than HA at 2 and 4 months No difference in functional outcomes between groups at any time
		HA 30	3	Hyruan Plus (HMWHA) (LG Life Sciences, Boston, USA)	9/21	67.5 ± 10.4	NA	I: 5(17.2) II:15 (51.7) III: 9 (31)				
T Kim 2023 [18]	Randomized double‐blind, multicenter, controlled trial	PN 30	3	Conjuran (PharmaResearch Co. Ltd, Gangneung, South Korea)	5/25	63.6 ± 6.7	NA	I: 0 II: 12 (40) III: 16 (53.3) IV: 2 (6.7)	VAS, WOMAC	2, 4 months	PN: Knee pain (*n* = 2) HA: Knee swelling (*n* = 1)	No difference in pain or functional outcomes between groups
		HA 30	3	Hyuran Plus (HMWHA) (LG Life Sciences, Boston, USA)	10/20	65.4 ± 8.2	NA	I: 1 (3.3) II: 8 (26.7) III: 18 (60) IV: 3 (10)				
W Kim 2023 [19]	Observational study	PN 546	5	Conjuran (PharmaResearch Co. Ltd, Gangneung, South Korea)	134/412	63.5 ± 9.1	NA	I: 72 (13.2) II: 249 (45.6) III: 225 (41.2)	VAS, CGI, PGI	3, 6 months	None	Significant pain improvement at 3 and 6 months
		No control	–	–	–	–	–	–				
Dallari 2018 [6]	Randomized, double‐blind, controlled clinical trial	PNHA 50	3	Condrotide Plus (Mastelli srl, Sanremo, Italy)	24/26	63.4 ± 6.5	28.1 ± 3.4	I–III	KSS, WOMAC	2, 6, 12 months	PNHA: None HA: None	Better total KSS and pain subscale with PNHA at 2, 6, 12 months compared to HA No difference in WOMAC between groups
		HA 50	3	Ialart (LMWHA) (Mastelli srl, Sanremo, Italy)	22/28	64.2 ± 5.1	28.1 ± 3.7	I–III				
Stagni 2021 [31]	Randomized, double‐blind, controlled trial	PNHA 39	3	Condrotide Plus (Mastelli srl, Sanremo, Italy)	24/26	63.4 ± 6.5	28.1 ± 3.4	I–III	WOMAC, KSS	24 months	PNHA: None HA: None	Significantly better KSS pain and WOMAC total score improvement in PNHA at 2 years
		HA 40	3	Ialart (Mastelli srl, Sanremo, Italy)	22/28	64.2 ± 5.1	28.1 ± 3.7	I–III				

Abbreviations: BMI, body mass index; BSV, bone structure value; HA, hyaluronic acid; HMWHA, high molecular weight hyaluronic acid; KOOS, Knee Injury and Osteoarthritis Outcome Score; KSS, Knee Society Score; LMWHA, low molecular weight hyaluronic acid; MMWHA, medium molecular weight hyaluronic acid; OA, osteoarthritis; PN, polynucleotide; PNHA, the combination of polynucleotides and hyaluronic acid; PROM, patient‐reported outcome measure; VAS, visual analogue scale; WOMAC, Western Ontario and McMaster Arthritis Index.

Within the meta‐analyzed cohort, four [10, 18, 25, 33] out of five studies reported study dropout. There were significantly more dropouts from the experimental group, with 18 patients (12.9%) compared to 6 (4.3%) in the HA group (*p* = 0.01). From the PN group, most dropouts were due to consent withdrawal (*n* = 5), unspecified reasons (*n* = 5), loss to follow‐up (*n* = 3), protocol violation with use of contraindicated medications (*n* = 3) and adverse effects not related to the product (*n* = 2).

Ten out of twelve studies included in the present systematic review and meta‐analysis utilized High Purification Technology (HPT™) PNs approved for intra‐articular use [6, 10, 11, 16, 18, 19, 25, 31, 33, 36]. One study used non‐HPT™ PNs [14], and the other did not list the PN product [22]. Within isolated PN treatments, four groups with a total of 92 patients received Condrotide (Mastelli srl, Sanremo, Italy) [10, 11, 33, 36] studies, and a total of 611 patients received Conjuran (PharmaResearch Co., Ltd, Gangneung, South Korea) [16, 18, 19, 25], and in one study, 51 patients received HP cell Vitaran J (BRPHARM Co., Wonju, South Korea) [14]. For PNHA, two studies injected a total of 50 patients with Condrotide plus (Mastelli srl, Sanremo, Italy) [6, 31]. Nine of the studies performed three weekly treatments [6, 10, 11, 16, 18, 22, 25, 31, 36], while the remaining three performed five injections [14, 19, 33]. The studies provided patient evaluation at 1 [10, 11, 16, 33, 36], 2 [10, 18, 22, 25, 33, 36], 3 [6, 11, 14, 19, 22], 4 [10, 18, 22, 25, 33, 36], 6 [6, 10, 11, 19], 12 [6] and 24 months [31]. VAS assessed pain, while function was evaluated with KOOS, KSS and WOMAC. The two RCTs comparing PNHA to HA [6, 31] were not included in the meta‐analysis due to differences in therapeutics as well as insufficient overlap in PROMs assessed and discrepancies in time points. Additionally, these studies [6, 31] included overlapping data, so a meta‐analysis of this treatment grouping would have been inappropriate.

### Publication bias

**Figure 2:**
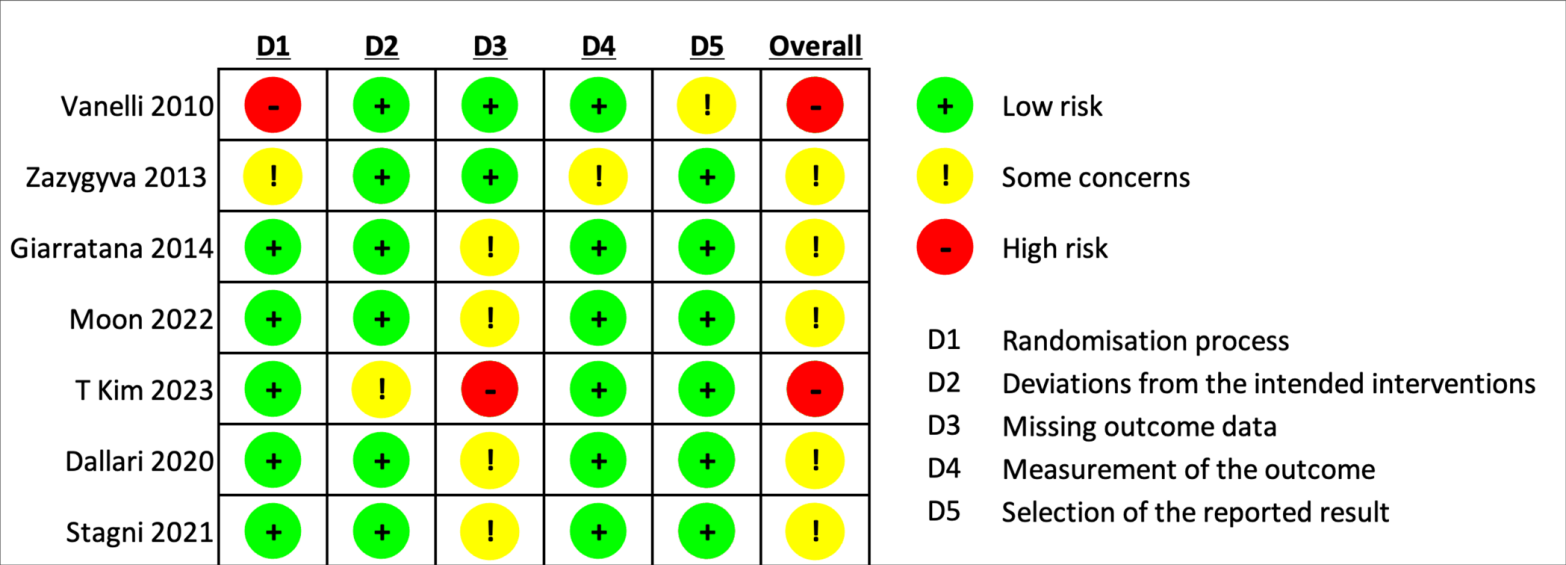
Risk of bias summary: Review authors' judgements for each risk of bias item for each included study. Each coloured cell represents the review authors' judgement for a specific domain: randomization process, deviations from intended intervention, missing outcome data, outcome measurement, and selection of reported results. Green = low risk, yellow = some concerns, red = high risk.

Out of seven RCTs reviewed, five had a moderate risk [6, 10, 25, 31, 36], and two had a high risk of bias [18, 33]. The most prevalent source of potential bias was missing outcome data, with four having some concerns [6, 10, 25, 31] and one having a high risk [18]. Figure 2 illustrates all methodological processes.

**Figure 3:**
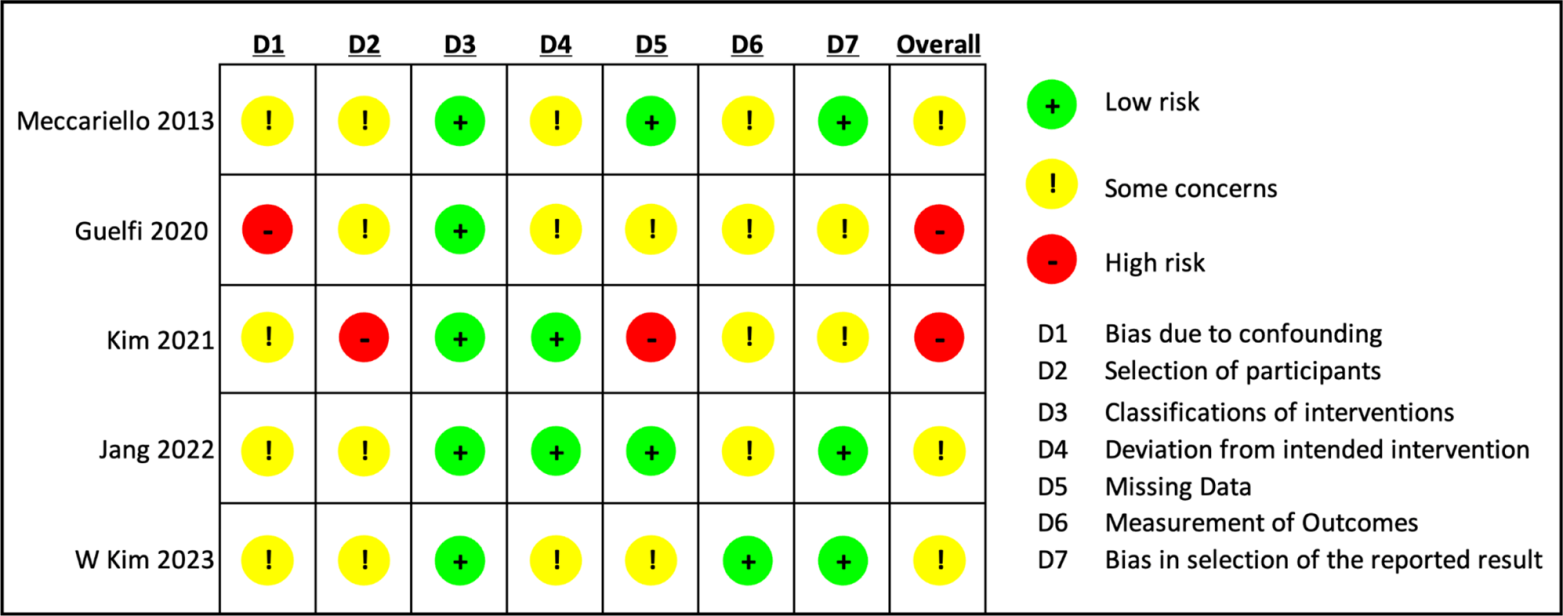
Risk Of Bias In Non‐randomized Studies of Interventions (ROBINS‐I) summary. Domains assessed include bias due to confounding (D1), participant selection, intervention classification, deviations from intended interventions, missing data, outcome measurement and selection of reported results. Risk levels are coded as follows: green = low risk, yellow = moderate risk and red = serious risk. ‘D1 bias due to confounding’ refers to systematic differences in baseline characteristics that may influence outcomes independently of the intervention.

The five remaining studies were evaluated for bias using the Risk Of Bias In Non‐randomized Studies of Interventions (ROBINS‐I). Three were deemed to be moderate risk [14, 19, 22], and two were high risk [11, 16]. Figure 3 describes each domain and its respective level of concern.

**Figure 4:**
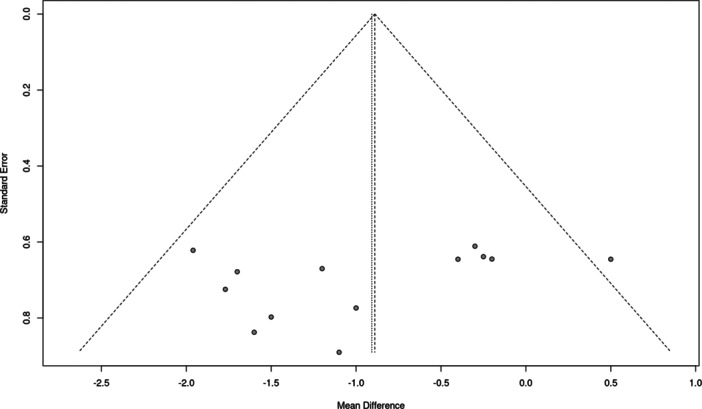
Funnel plot of pain (VAS) data from randomized controlled trials. The *x*‐axis shows the effect size estimate (standardized mean difference), and the *y*‐axis shows the study precision (standard error). The diagonal lines represent the 95% confidence intervals. Asymmetric plot distribution from the central vertical line (overall effect) suggests publication bias. VAS, visual analogue scale.

Since all studies included within the meta‐analysis reported VAS, pain outcome data were utilized to assess publication bias using a funnel plot. There was moderate data heterogeneity (*I* ^2^ = 51%), which indicates an asymmetric distribution of data (*p* < 0.01) and, ultimately, a potential risk of bias (Figure 4).

### PNs

#### Pain

**Figure 5:**
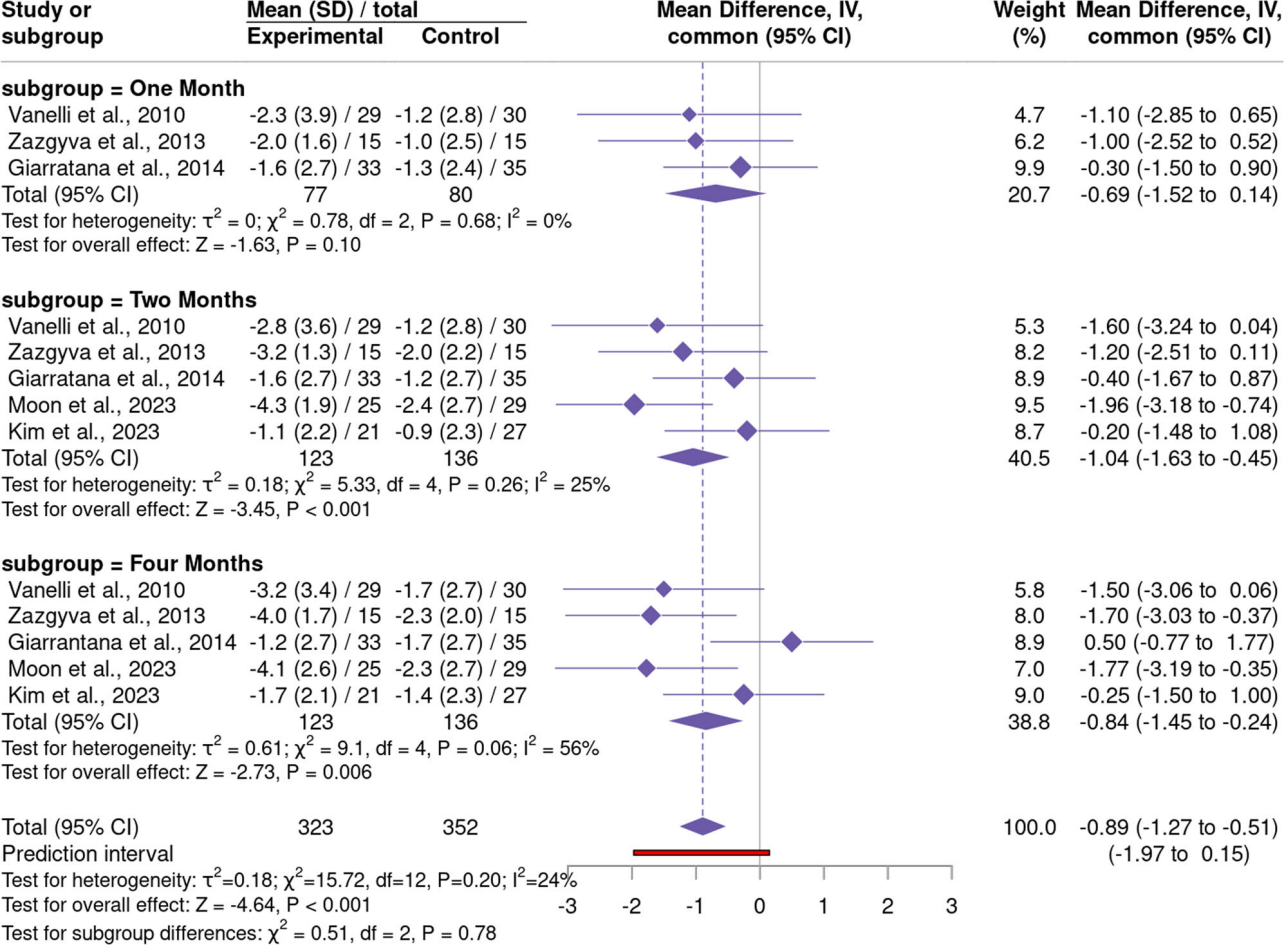
A forest plot comparing pain (VAS) scores between polynucleotide and hyaluronic acid groups. Negative values favour polynucleotide injections. CI, confidence interval; SD, standard deviation; VAS, visual analogue scale.

Of the five studies included in the meta‐analysis, three evaluated pain at 1 [10, 33, 36], five at 2 [10, 18, 25, 33, 36] and five at 4 months [10, 18, 25, 33, 36]. The pooled analysis demonstrated nearly a one‐point relative reduction in pain scores for PN treatments over HA (MD = −0.89 [−1.27 to −0.51], *p* < 0.001). Initial follow‐up data approached significance at 1 month (*p* = 0.103). However, the effect size grew at 2 (MD = −1.04 [−1.63 to −0.45], *p* = 0.0006) and 4 months post‐injection (MD = −0.84 [−1.45 to −0.24], *p* = 0.006) (Figure 5).

Among the non‐RCT studies, most found early pain improvement. Wan‐Ho Kim and Jang et al. evaluated a 5‐shot treatment regimen and noted significant improvements (30–35 point reduction out of 100) in non‐weighted VAS that persisted for at least 3–6 months [14, 19]. Kim et al.'s large observational study of 546 patients demonstrated greater than 50% pain reduction in all OA severities evaluated (KL I–III), with slightly better pain improvement in mild compared to moderate OA (−60.7% vs. −51.5%) [19]. Ji Yeong Kim et al.'s small cohort of five patients in PN and HA groups recorded a 52‐point reduction (64–12) in VAS scores in 1 month in PN compared to no change in the HA group (44–46) [16]. However, pain outcomes from Meccariello's study were not as impressive. Their patients did not experience any difference in pain for 6 months, although this was only a 1.5‐point difference on a 10‐point scale [22].

### Function

**Figure 6:**
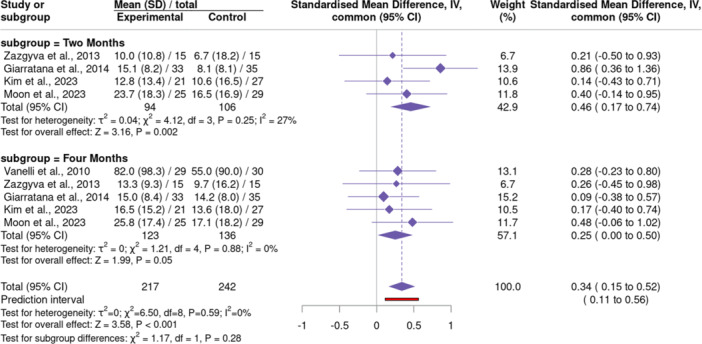
A forest plot comparing functional outcomes (KOOS, KSS and WOMAC) between polynucleotide and hyaluronic acid groups. Positive values favour polynucleotide injections. CI, confidence interval; KOOS, Knee injury and Osteoarthritis Outcome Score; KSS, Knee Society Score; SD, standard deviation; WOMAC, Western Ontario and McMaster University Osteoarthritis Index.

Multiple functional PROMs were assessed, including KOOS [10, 33], KSS [36] and WOMAC [18, 25]. Of the five RCTs that included PROMs, four had enough overlapping data for meta‐analysis at 2 months [10, 18, 25, 36] and five at 4 months [10, 18, 25, 33, 36] of follow‐up. The pooled meta‐analysis demonstrated better functional outcome scores among the PN group compared to HA (standardized mean difference [SMD] = 0.34 [0.15–0.52], *p* < 0.001). The majority of this effect was within the 2‐month subgroup analysis, which revealed nearly a 0.5 SD improvement in the experimental group relative to control (SMD = 0.46 [0.17–0.74], *p* = 0.002). Functional improvement at 4 months approached statistical significance (SMD = 0.25 [0.00–0.50], *p* = 0.05) (Figure 6).

The four remaining non‐RCTs comparing PN to HA evaluated functional outcome scores. Meccariello et al. were the only ones to use KOOS. The group found no difference between PN and HA at 1, 3 or 6 months [22]. Kim et al. and Guelfi also found no difference in WOMAC scores at one month compared to controls [11, 16]. However, Guelfi's study compared historical controls rather than a prospective control group. Jang et al.'s prospective cohort noted a significant improvement in WOMAC scores at the final injection and 3 months post‐treatment. However, there was no control to compare [14].

### PNHA

#### Pain

Reported pain outcome measures were conflicting. Dallari et al. found significant improvements in pain with the KSS subscale scores between PNHA and HA‐treated patients at 2 and 12 months but revealed no difference in WOMAC pain scores at any time point [6]. Stagni et al. published a 2‐year follow‐up study with the same patient population. The group found no overall difference in pain with the long‐term evaluation. However, the group noted that patients with moderate OA (KL Grade III) receiving PNHA tended to have better pain improvement compared to HA‐treated controls with the same severity of OA, as well as patients with less arthritic knees from either treatment group (2‐point vs. 1‐point improvement) [31].

#### Function

The two included studies evaluated function with WOMAC and KSS scores. Dallari et al. found early and persistently better improvements in KSS scores at 2, 6 and 12 months with PNHA over HA, but no intergroup difference in composite WOMAC scores at any time point [6]. Interestingly, Stagni et al. revealed significantly better WOMAC scores with those receiving PNHA than HA after 2 years (18‐point vs. 6‐point improvement), but they could not produce similar findings with KSS [31].

#### Adverse events

Eleven studies assessed adverse events [6, 10, 11, 16, 18, 19, 22, 25, 31, 33, 36]; however, only nine had a HA control comparison group [6, 10, 16, 18, 22, 25, 31, 33, 36]. Among the studies with a control, nine reported adverse events were observed in 247 PN patients (3.6%), and five were reported in 261 HA patients (1.9%). These side effects included transient knee pain (PN and PNHA = 7, HA = 3) and knee swelling (PN and PNHA = 1, HA = 1), which spontaneously resolved. There was no significant difference in adverse events between experimental and HA control groups (RR = 1.97, 95% CI = 0.72–‐5.37, *p* = 0.187). No serious complications, including intra‐articular infections, were reported.

### Biomarker measurement

Three studies [6, 10, 14] evaluated pro‐inflammatory and catabolic markers before and after treatment with PNs. Two of these studies assessed synovial fluid levels [6, 14]. Dallari et al. suggested that treatment with PNHA promoted a slightly less inflammatory state, with a greater reduction in MMPs and TNF‐α than HA alone [6]. The single‐arm study by Jang et al. measured IL‐6, IL‐10 and TNF‐α at baseline and after the fifth PN injection. The group found no differences in cytokine levels following treatment [14]. Giarrantana et al. assessed Cartilage Oligomeric Matrix Protein (COMP) serum levels before and at 1 and 6 months post‐treatment. The group found an earlier reduction of COMP levels with HA compared to PN‐treated patients, but had similarly lower levels by 6 months [10].

### NSAID use

Six studies evaluated concurrent analgesic consumption [10, 18, 22, 31, 33, 36]. Overall, there were no significant differences in reported medication use between treatment and control groups at any time point; however, three groups reported earlier reductions in medication use in patients receiving PNs [10, 18, 36].

### Imaging changes

One study evaluated structural changes to the knee joint following PN injection [14]. Using an AI algorithm‐based texture analysis of bone structure values (BSVs), Jang et al. noted improvements in the microstructure of the middle and deep subchondral bone layers within the lateral femoral condyle 3 months after treatment. The authors postulated that this finding could translate to signs of subchondral bone remodelling. However, there was no control group to compare subchondral bone modifications.

## DISCUSSION

The key finding of this systematic review is that PNs demonstrate statistically significant improvements in pain relief and functional outcomes compared to HA after a period of two to four months. Similar results were noted in non‐RCTs; however, functional measures did not show statistically significant differences between the two groups. The combined therapy of PNs and HA also appears to reduce pain and enhance function; however, the available data are limited, making it difficult to draw strong conclusions. Adverse events between the different PN formulations and HA were comparable.

We demonstrated that PNs provided a roughly one‐point better improvement in pain scores than HA. Although this improvement relative to the control group was statistically significant, its clinical relevance may be questionable. Nevertheless, the extent of relief from baseline measurements appears to be clinically meaningful. In research settings, multiple metrics, including the minimally clinically important improvement (MCII), serve as benchmarks for determining clinically significant improvements. Silva et al. reported that the MCII for VAS in individuals with knee OA receiving nonoperative management is 1.99 out of 10 [30]. Among the meta‐analyzed studies, this threshold was surpassed in two out of three at 1 month [33, 36], and three out of five at 2 and 4 months [25, 33, 36]. Moreover, most control subjects were treated with high molecular weight HA (HMWHA), which has demonstrated stronger pain relief and functional improvement than low molecular weight HA (LMWHA) [27]. This effectiveness may be attributed to HMWHA's properties, which more closely resemble healthy synovial fluid than LMWHA [26].

Two studies in this review evaluated the impact of OA severity on pain response after PN injection [19, 31]. Interestingly, both studies demonstrate similar improved outcomes regardless of the extent of joint disease. Kim et al.'s large patient cohort received a five‐shot regimen of PNs and experienced at least a 50% reduction in pain at all time points, irrespective of OA severity. However, those with milder OA did show slightly better pain improvement than those with moderate OA [19]. Stagni's group demonstrated that patients with KL Grade III who received PNHA had better pain relief than controls with the same degree of OA after two years of follow‐up [31]. These findings contrast with the general understanding regarding HA and most other treatments, which typically show a higher risk of treatment failure as the KL grade increases to III or IV [7, 28].

This is not the first study to synthesize clinical data on PNs. In 2019, Kim et al. performed a systematic review and meta‐analysis of five studies (three PNs and two PNHAs) [6, 10, 33, 35, 36]. They concluded that PNs improved pain relief after 1–2 months but did not provide any functional benefit [17]. The updated analysis includes data from eight newer studies—seven focusing on PNs and one on PNHA [11, 14, 16, 18, 19, 22, 25, 31]—and suggests more persistent pain relief lasting at least 4 months. Some studies indicate that analgesia may last over a year after the injection [6, 31]. Additionally, our meta‐analysis on functional outcomes yielded better results with PNs compared to HA injections. The current review also differs by distinguishing PNHA treatments from strictly PN in the analysis. The earlier review included PNHA data from the studies by Yoon et al. and Dallari et al. [6, 35] in their meta‐analysis, which may have influenced their conclusions. Moreover, Yoon et al.'s study was excluded from our review as the group utilized a therapy that is not approved for intra‐articular use (Placentex, Mastelli srl, Sanremo, Italy) [35]. Furthermore, we have discussed non‐RCTs to present a more comprehensive view of the available evidence. While these articles also reported positive outcomes for pain relief, functional improvement was not consistently observed.

The current review suggests the possible additive effect of combining HA with PNs for improved pain and function. A recent in vitro study supports this claim. Guizzardi et al. found that adding HA to PN for fibroblast cell cultures enhanced cell proliferation and viability by an additional 20% beyond the mitogenic effect observed with low‐dose PNs [12]. Given the limited evidence on the combined therapy, further clinical studies are needed to evaluate the differences in pain and function between PN and PNHA in human subjects.

In addition to knee OA, several small studies have evaluated PNs for hip, ankle and temporomandibular joint (TMJ) OA. Migliore et al. followed 43 patients with KL Grade II–III hip OA, who received biannual injections of PN (Condrotide, Mastelli srl, Sanremo, Italy) over a three‐year period. The group demonstrated a 50% reduction in pain at 6 months, which persisted throughout the study. Function was assessed using the Lequesne Index, and improvements were observed at 6 months, with additional gains noted at 2 and 3 years [24]. Guelfi et al. studied 56 patients with ankle OA graded from I to IV. Patients received three weekly injections of PN (Condrotide, Mastelli srl, Sanremo, Italy) and were followed for 6 months. Authors noted that Foot and Ankle Outcome Scores nearly doubled at 1 month post‐injection and persisted for 6 months [11]. Cenzato et al. compared the effectiveness of jaw exercises to Poliart (PNHA, Mastelli srl, Sanremo, Italy) injection for degenerative TMJ pathology in a study involving 60 patients (30 in each group). The study revealed significantly better pain improvement in the PNHA group at three months, although there was no difference in jaw opening angle between the two groups [4]. Cumulatively, these reports are promising for PNs as a new, versatile, non‐cellular therapy for various joint pathologies, including those outside of knee OA.

As mentioned earlier in the study, PNs are not without potential adverse effects. Most reported adverse events for intra‐articular injections were transient pain or swelling that occurred shortly after treatment. Fortunately, none of the papers described long‐lasting discomfort, systemic symptoms, or joint infections. Although PN‐treated patients reported these more frequently than those with HA, there was no statistically significant greater risk associated with PN. Orthopaedic literature on PNs is limited, and no absolute contraindications or long‐term effects have been published. However, one should avoid recommending PN injections for patients with a fish protein allergy, as these injections are derived from trout or salmon. Like other intra‐articular products, PNs should be contraindicated in those with concerns for septic joint or skin breakdown near the injection site.

From this review, we have several clinical recommendations. The meta‐analysis suggests that pain and functional outcomes are better in the short term with PNs compared to HA, indicating that PNs may be a more effective initial viscosupplement. The potential for PN and PNHA to deliver both mechanical and biological benefits supports their further evaluation as a meaningful advancement in OA treatment. Additionally, PNs may be a preferable option for patients with known allergies to HA products or additives. PN therapy could also be considered for patients who have recently not responded well to HA, or it may serve as an adjunct to HA treatment. Furthermore, PNs alone or PNHA formulations may be more appropriate for patients with moderate OA (KL III) than HA. However, more research is needed to assess the long‐term effects of PN and PNHA treatments on pain and function compared to current standards of care. These findings highlight the clinical need for alternatives to HA, particularly for patients who have inadequate responses or contraindications to HA. Future research should also consider other clinical metrics, such as the time to knee arthroplasty.

### Limitations

The current study has several limitations. First, the variability in data from different PROMs (such as WOMAC, KSS and KOOS) and varying evaluation time points made statistical analysis challenging and difficult to interpret, which ultimately affected the statistical power. Additionally, the studies examined a range of PN‐HPT and HA formulations, as well as differing injection series (three vs. five treatments), which further complicated comparisons. Moreover, the post‐injection protocols varied between studies, with some allowing NSAIDs/Tylenol while others prohibited them. Furthermore, inconsistent reporting of knee OA severity may have contributed to differences in pain and functional responses between studies.

Furthermore, several potential biases were identified in the included studies that could affect data interpretation and presentation. A primary concern in the RCTs was loss to follow‐up, although the overall dropout rate was low. Additionally, funnel plot analysis indicated data heterogeneity, which may suggest bias in data collection and analysis. In contrast, the non‐RCTs exhibited more questionable features, including issues with confounding, problems with participant selection, and missing data. Funding also raises potential concern; the majority were either industry‐funded or supported by companies such as Mastelli srl [6, 11, 31, 33], PharmaResearch Co. Ltd [16, 18, 19, 25] or BRPHARM Co [14], or were conducted as a post‐marketing trial [10]. It's also worth noting that the principal investigator for this review is a paid consultant for Mastelli srl, which may introduce further bias. Another consideration is the quality of evidence from publications released in non‐indexed journals [22, 36], which could compromise reliability. Finally, the findings from these studies may not be widely applicable, as injectable PNs are not commercially available in all countries, including the United States.

## CONCLUSION

Intra‐articular PN injections offer a safe and efficacious treatment option for symptomatic knee OA. In the first 4 months following treatment, they may provide greater pain relief and improved function than HA. Additionally, limited data suggest an additive benefit for pain and function with a combination therapy of PNs and HA. However, the variability in PROMs and different PN formulations highlights the need for additional research to better understand the clinical benefits of this new treatment compared to current standards of care.

## AUTHOR CONTRIBUTIONS

Joseph Elphingstone was involved in study conceptualization, design, data collection, statistical analysis, drafting and revising the manuscript. Michael Bowler was involved in data collection, presentation and manuscript drafting. Pietro Conte was involved in conceptualizing the study and editing the manuscript. Giuseppe Anzillotti was involved with conceptualizing the study and editing the manuscript. Elizaveta Kon was involved in reviewing and editing the manuscript, as well as project supervision.

## CONFLICT OF INTEREST STATEMENT

Elizaveta Kon is a paid consultant for Mastelli srl. The remaining authors declare no conflicts of interest.

## ETHICS STATEMENT

The ethics statement is not available.

## Data Availability

Data available on request from the corresponding author.
